# Prospective controlled study comparing 5.5-mm long implants with longer implants to support fixed partial prosthesis in the premolar-molar regions: 12 months follow-up

**DOI:** 10.1038/s41405-025-00392-y

**Published:** 2025-12-17

**Authors:** Eduardo Anitua, Adriana Montalvillo, Mohammad Hamdan Alkhraisat

**Affiliations:** 1 University Institute for Regenerative Medicine and Oral Implantology—UIRMI (UPV/EHU-Fundación Eduardo Anitua), Vitoria, Spain; 2 BTI Biotechnology Institute, Vitoria, Spain; 3 Department of Cellular Biology and Histology, Faculty of Medicine and Nursing, Universidad del País Vasco/Euskal Herriko Unibertsitaea (UPV/EHU), Leioa, Spain; 4 Oral and Maxillofacial Surgery, Oral Medicine and Periodontics Department, Faculty of Dentistry, University of Jordan, Amman, Jordan

**Keywords:** Dental implants, Fixed prosthodontics

## Abstract

**Aim:**

This prospective controlled clinical study aimed at evaluating the performance of 5.5 mm-long implants to support two-unit fixed prosthesis in the posterior regions of the mandible and the maxilla.

**Materials and methods:**

A total of 44 patients (30 females and 14 males) were included and received 51 test implants (5.5 mm in length) and 51 control implants (≥6.5 mm in length) to support two-unit fixed partial prosthesis in the mandible and the maxilla. Intermediate abutment was used in all cases. The patients were followed for 12 months. Changes in the marginal bone loss, implant survival, and prosthetic complications were compared between the two groups.

**Results:**

The patient’s mean age was 64 years (range: 42–48 years). All the implants in the test group were placed at the molars site compared to 26 implants in the control group (*p* < 0.001). Bone type (*p* = 0.224) and insertion torque (*p* = 0.671) were similar between the groups. None of the implants failed. The changes in marginal bone level at mesial (median: 0.3 mm; range: −1.3 to 2.9 mm for control, and 0.2 mm; −0.7 to 1.6 mm for test; *p* = 0.138) and at distal (median: 0.3 mm; range: −1.3 to 1.5 mm for control, and 0.2 mm; −1.2 to 1.4 mm for test; *p* = 0.0633) were similar between the study groups. The prostheses were free of technical complications.

**Conclusions:**

This prospective controlled clinical study supports the use of 5.5-mm long implants in the context of fixed partial prosthesis and posterior regions. No statistically significant differences have been observed in marginal bone stability, implant survival and prosthetic complications compared to longer implants. Within the limits of this study, 5.5 mm implants appear to be a predictable option for fixed posterior partial dentures.

## Introduction

Short dental implants have emerged as an alternative to alveolar bone augmentation techniques when insufficient residual alveolar height is available to place longer implants [1–8]. Comparing the performance of short dental implants with vertical bone augmentation and the placement of longer implants, no significant differences have been observed in terms of implant survival or peri-implant marginal bone stability [3–8]. However, lower occurrence of biological complications has been observed [9]. The placement of short implants in atrophic mandible has been proved to be an alternative to vertical augmentation surgeries, demonstrating a lower occurrence of post-surgical complications and less marginal bone loss after 5 years of follow-up [10–12]. Moreover, short implants could avoid the complexity of interventions for inferior dental nerve repositioning while demonstrating similar results in clinical practice [13]. The good performance of short implants in the mandible has been corroborated in a recent systematic review [8].

In this context, extra-short implants (<6.0 mm) have also been proposed as an alternative for the rehabilitation of extremely vertically resorbed ridges [6]. Systematic reviews with meta-analyses comparing extra-short implants with short or standard implants have documented the absence of significant differences in terms of survival rates and marginal bone stability in the short and medium term [6, 14, 15]. Twenty-eight 5.5-long implants have been placed in alveolar ridges with a residual height of 4.5 ± 0.6 mm [16]. The survival rate has been 96% after 5 years of follow-up. The mean marginal bone loss has been 0.3 ± 0.7 mm. Moreover, the assessment of 100 4.0-mm long implants in 32 patients has indicated a cumulative survival rate of 92% and a marginal bone loss of 0.56 after 2 years of follow-up [17]. Another prospective comparative study has reported the absence of statistically significant differences between implant lengths of 4–6 mm versus implant lengths of 8–10 mm after 1 year of follow-up [18]. Torassa et al. [19] have reported the results of 4 mm-long implants splinted to 8 mm-long implants in terms of survival, marginal bone loss, and resonance frequency analysis. A total of 22 implants (11 extra-short implants and 11 short implants) have been placed in 11 patients and have been followed for 2 years. None of the implants studied failed during the follow-up period, and the marginal bone loss was 0.3 mm at the end of the study. Moreover, osseointegration increased progressively.

However, the allocation of short implant and comparator into different patients would introduce biological variations that act as confounding factors in the inter-subject comparisons. The placement of short implants and the comparator in the same patient would enable intra-subject comparison and minimize the risk of biological variability between subjects. This prospective controlled clinical study provides a comparison of 5.5mm-long implants with longer implants in the same patients and being restored by the same prosthesis. All the prostheses have been fixed partial denture supported by 2 implants. The study hypothesis has been that both implant types would similarly perform, with absence of statistically significant differences, regarding marginal bone stability, implant survival and prosthetic complications.

## Materials and methods

The manuscript was prepared following the guidelines of strengthening the reporting of observational studies in epidemiology [20]. This clinical research was an observational prospective study comparing the performance of implants less than 6.5 mm in length (test group) with longer implants (control group). Both implant types were splinted in the same prosthesis, comparing intra-subject behavior and eliminating confounding factors related to biological factors. To reduce inter-observer variability, the measurement of marginal bone level from the radiographic record was performed by the same researcher. Furthermore, the implants in this study presented the same surface characteristics (UnicCa^®^ surface, BTI Biotechnology Institute, Vitoria, Spain).

The clinical research was conducted by adhering to the ethical principles of the Declaration of Helsinki and was approved by the Research Ethical Committee of University Hospital of Álava (FIBEA-02-EP/20/Extracortos). Prior to participation, all individuals received information on the study’s procedures and potential risks, and gave their written, informed consent.

Subjects were recruited in a single private clinical center (Vitoria, Spain) between June 2021 and July 2022. They were included in the study as they visited the participating center and if they met all the inclusion criteria and met none of the exclusion criteria.

The inclusion criteria were:

- Age ≥ 18 years.
- Treatment planning the aimed to restore missing teeth with a fixed multiple implant-supported prosthesis and the insertion of implants <6.5 mm in length and implants ≥6.5 mm in length.
- The insertion of a total of 2–4 implants per prosthesis.
- Availability of a previously taken Cone-Beam Computed Tomography (CBCT) scan.
- Availability for observation during the follow-up period.
- Delivery of signed informed consent.

The exclusión criteria were:

- Smoking more than 10 cigarettes/day.
- Case restoration with a complete or single-unit prosthesis.

### Procedures

The selection of implant dimensions (diameter and length) was determined after analyzing the residual alveolar bone in a CBCT scan, following the daily clinical practice of the clinical center. No randomization was performed. Blinding the surgeon and the clinical evaluator to the implant type could not be possible due to the differences in implant length between the study groups. The researcher who performed the data analysis was blinded to the study groups.

After the administration of local anesthesia, a full thickness flap was elevated to expose the underlying alveolar bone. Implant site was prepared following low-speed drilling protocol and the implant was initially inserted with a surgical motor at a torque of 25 Ncm [21, 22]. The implant was finally seated with a torque wrench, and the insertion torque was annotated. Immediate loading was decided if the implant was placed in bone type I, II, or III, and the insertion torque was higher than 25 Ncm. Otherwise, two-stage surgical procedure was performed. For implant loading, an intermediate abutment with a height ≥2 mm (Multi-Im, BTI Biotechnology Institute, Vitoria, Spain) was connected to the dental implant and an impression was made. The provisional prosthesis was then prepared by using the articulated titanium bar system (BTI Biotechnology Institute, Vitoria, Spain) that was veneered in resin. The ceramic-veneered, screw-retained final prosthesis featured a CAD/CAM-produced metal substructure to establish a mutually protected occlusal scheme. Worth mentioning the application of fraction 2 (F2) of the plasma rich in growth factors (PRGF), in a liquid state, at the implant site just before insertion. To obtain the PRGF, 9 mL tubes containing 3.8% sodium citrate (KMU 15, BTI Biotechnology Institute, Vitoria, Spain) were used for blood extraction [23, 24]. The tubes were then centrifugated at room temperature according to the manufacturer instructions and processed to obtain F2 of the PRGF that contained the 2 mL of the plasma column just above the buffy coat.

### Study variables

Implant survival was defined as the presence of the dental implant in the patient mouth after 1 year of insertion. Changes in marginal bone level were defined as the changes in the distance between the implant platform and the most coronal contact point at the implant-bone interface in the period between implant loading and 1 year later. For that, periapical radiographs were obtained with parallel technique and using Rinn XPC-ORA radiographic positioner (Dentsply Sirona, Charlotte, NC, USA). The radiographs were assessed in DIGORA® for Windows software (version 2.9.113.490, Soredex, Milwaukee, WI, USA) and were calibrated by the known implant length. The measurements were performed at the mesial and distal aspects of the dental implant. Prosthetic complications were assessed by the frequency of occurrence of technical complications (porcelain fracture, screw loosening, screw fracture, and prosthesis fracture) during the follow-up.

### Sample size

In a previous study [25], a difference in marginal bone loss of 0.19 mm was observed with a standard deviation of 0.231 mm. With a1:1 ratio in the distribution of control and test groups, a Type I error (α) of 0.01, and a statistical power (1-β) of 0.9, 45 dental implants per group were required to reject the null hypothesis that there were no differences between the study groups regarding marginal bone loss. Considering a 15% loss to follow-up rate, 51 implants per group were needed.

### Statistical analysis

A description of the population was conducted using the demographic and clinical variables. For this purpose, continuous variables were expressed as mean with standard deviation and range, while categorical variables were expressed as absolute and relative frequency. The normality of the variables was assessed by the Shapiro-Wilk test. Statistical differences between continuous variables were assessed using the paired Student’s *t*-test (bone density, distal marginal bone level (loading, and 1-year) and changes in distal marginal bone level) or Wilcoxon test (insertion torque, follow-up time, mesial marginal bone level (loading, and 1-year) and changes in mesial marginal bone level). Differences between categorical variables were assessed using the Chi-square test. The cumulative survival of the implants and prostheses was assessed with Kaplan-Meier curves, and the effect of length was analyzed using the Log-rank statistic. In all cases, differences with a *p* < 0.05 were accepted as statistically significant. The statistical analysis was performed using SPSS Statistics version 15.0 (IBM, Armonk, NY, US).

## Results

**Fig. 1:**
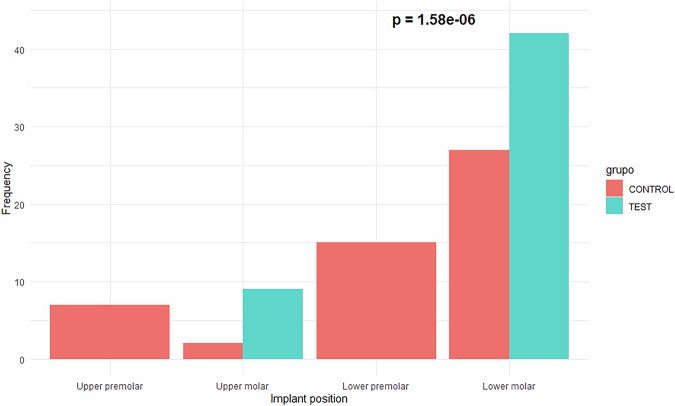
Anatomical location. Implant position in the control and test groups. Chi-squared test.

Forty six patients were screened for eligibility to participate in this study, however 2 patients were excluded as they could not comply with the scheduled visits. A total of 44 patients participated, contributing with 51 control implants and 51 test implants. 30 patients were females and 14 were males. The patient’s mean age was 64 years (range: 42–82 years). All the implants were placed in the premolar and molar regions of the maxilla (18 implants) and the mandible (84 implants). Figure 1 illustrates the distribution of the anatomical positions of the dental implants. The differences between the study groups were statistically significant. All implants in the test group were placed in the molar region.

**Fig. 2:**
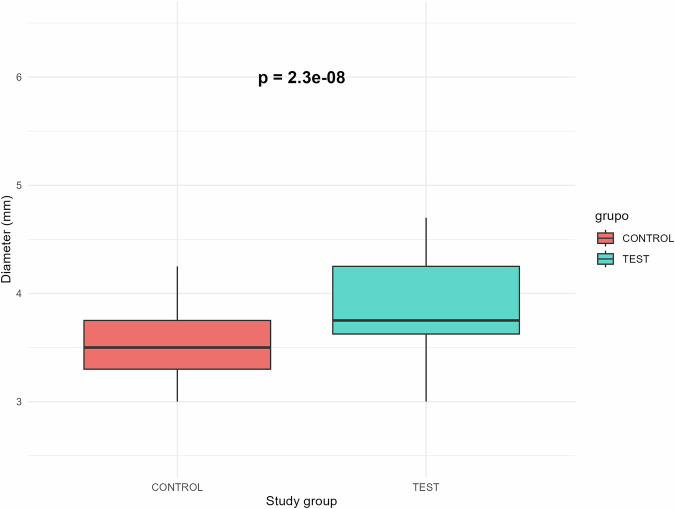
Implant dimensions. The diameter of the dental implants in the control and test groups.

The implants were placed according to standard clinical practice; that is, shorter implants were used when the available bone height precluded the placement of longer implants. All test implants had a length of 5.5 mm. In the control group, 32 implants were 6.5 mm long and 19 were 7.5 mm long. The diameters of the test implants were significantly larger than those of the control implants (Chi-squared test, *p* < 0.001). The implants in the control group had diameters ranging from 3.0 to 4.25 mm, while those in the test group ranged from 3.0 to 4.7 mm (Fig. 2).

Table 1 shows date relevant to implant placement. The insertion torque had not differed significantly between the study groups (Wilcoxon test, *p* = 0.671). The median values were 40 and 45 N.cm for the control and test groups, respectively. The bone density significantly differed between the two groups (paired Student’s *t* test, *p* = 0.02), being denser in the control group (mean: 694.1 units) than the test group (mean: 624.5 units). However, the distribution of the bone type between the study groups was not statistically different (Chi square test, *p* = 0.221). All the bone types in the control groups were between bone type I and type III, whereas bone type IV was only present in the test group. As both implant types supported the same prosthesis, the implant loading was identical between the study groups. Delayed loading was performed in 14 of the 18 implants in the maxilla and in 18 of the 66 implants in the mandible.

**Table 1:** Placement and loading type of dental implants in control and test groups.

Characteristic	CONTROL *N* = 51^*1*^	TEST *N* = 51^*1*^
Insertion torque (N.cm)		
Mean	43.8	42.4
Median	40.0	45.0
Min–Max	10.0–70.0	5.0–65.0
Bone type		
Tipo I	6 (12%)	5 (9.8%)
Tipo II	30 (59%)	26 (51%)
Tipo III	15 (29%)	16 (31%)
Tipo IV	0 (0%)	4 (7.8%)
Bone density		
Mean	694.1	624.5
Median	700.0	600.0
Min–Max	350.0–1150.0	200.0–1100.0
Loading type		
Delayed	16 (31%)	16 (31%)
Immediate	35 (69%)	35 (69%)
^*1*^n (%)		

None of the dental implants failed during the observation period. Table 2 summarizes the data on follow-up times and marginal bone levels around the dental implants. The follow-up duration did not differ significantly between the study groups (Wilcoxon test, *p* = 0.884). However, the marginal bone level differed significantly between the groups at both the time of loading and at the final radiographic evaluation.

**Table 2:** Peri-implant bone level changes by implant type and loading protocol.

Characteristic	CONTROL *N* = 51	TEST *N* = 51
Marginal bone level at loading (mesial)		
Mean	−1.2	−0.7
Median	−1.1	−0.7
Min–Max	−4.0 to 0.7	−3.7 to 0.8
Marginal bone level at loading (distal)		
Mean	−0.3	−0.8
Median	−0.3	−0.8
Min–Max	−2.1 to 1.0	−2.8 to 0.6
Marginal bone level at 1 year (mesial)		
Mean	−0.8	−0.4
Median	−1.0	−0.4
Min–Max	−3.7 to 1.2	−2.4 to 0.8
Marginal bone level at 1 year (distal)		
Mean	0.0	−0.7
Median	0.0	−0.7
Min–Max	−1.8 to 1.8	−1.9 to 0.6
Change in marginal bone level (mesial)		
Mean	0.4	0.2
Median	0.3	0.2
Min–Max	−1.3 to 2.9	−0.7 to 1.6
Change in marginal bone level (distal)		
Mean	0.3	0.1
Median	0.3	0.2
Min–Max	−1.3 to 1.5	−1.2 to 1.4
**Immediate loading**		
Change in marginal bone level (mesial)		
Mean	0.4	0.2
Median	0.3	0.2
Min–Max	−0.6 to 2.9	−0.7 to 1.6
Change in marginal bone level (distal)		
Mean	0.4	0.2
Median	0.3	0.2
Min–Max	−1.3 to 1.5	−0.8 to 1.4
**Delayed loading**		
Change in marginal bone level (mesial)		
Mean	0.3	0.1
Median	0.3	0.0
Min–Max	−1.3 to 2.7	−0.3 to 1.2
Change in marginal bone level (distal)		
Mean	0.2	−0.1
Median	0.3	−0.2
Min–Max	−1.0 to 1.1	−1.2 to 0.9

At the mesial side, the control and test implants were subcrestally located, with the implant platforms located 0.7 mm and 1.1 mm below the marginal bone level, respectively (Wilcoxon test, *p* = 0.003). At the last radiograph, the implant platforms were located 1.0 mm and 0.4 mm below the marginal bone level in the control and test groups, respectively (Wilcoxon test, *p* = 0.02).

On the distal side, the implant platforms were located 0.3 mm and 0.8 mm subcrestally in the control and test groups, respectively (paired Student’s *t*-test, *p* < 0.0001). At the last radiograph, these distances were 0.0 mm and 0.7 mm, respectively (paired Student’s *t*-test, *p* = 0.0000).

**Fig. 3:**
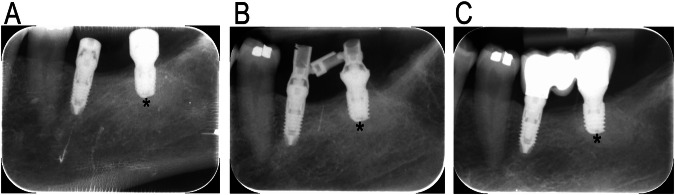
Clinical case. Periapical radiographs showing the marginal bone level at insertion (**A**), loading (**B**) and 12-month (**C**). (*) denotes the test implant.

Despite these positional differences, the change in marginal bone level over time did not differ significantly between the study groups at either the mesial (Wilcoxon test, *p* = 0.138) or distal sides (paired Student’s *t*-test, *p* = 0.0633) (Fig. 3).

Furthermore, Table 2 presents the changes in marginal bone level according to the loading protocol. Under the delayed loading protocol, the changes in mesial marginal bone levels for both the test and control implants were not statistically significant (Wilcoxon test, *p* = 0.074). Likewise, changes in distal marginal bone levels showed no significant differences between the two groups (paired Student’s *t*-test, *p* = 0.149).

Similar findings were observed in the immediate loading protocol, where no statistically significant differences were found in mesial (Wilcoxon test, *p* = 0.182) or distal (paired Student’s *t*-test, *p* = 0.234) marginal bone level changes between the test and control implants.

## Discussion

The current prospective clinical study has compared extra-short and longer implants in a clinical setting that has eliminated variability in healing time, implant loading and prosthetic rehabilitation between the study groups. The null hypothesis has been accepted due to the absence of statistically significant differences in the change in the marginal bone level, implant survival and prosthetic complications in the context of 1 year of follow-up.

Compared to bone augmentation procedures, the placement of short implants (≤6 mm) in pristine bone can reduce treatment cost, treatment time and morbidity, thereby enhancing patient satisfaction [6, 14, 26, 27]. In the current study, both implant types have been placed in pristine bone and have shown no statistically significant differences in the variable of changes in marginal bone level. A recent meta-analysis has compared (≤6 mm) implants and long implants (≥10 mm) that were placed in pristine bone [6]. There has been absence of statistically significant differences of marginal bone loss at 1-year of follow-up (mean difference: −0.04 mm; 95% confident interval (CI): −0.08 to 0.17). Similarly, there has been absence of statistically significant differences in biological complications [6]. These data agree with the finding of the current study that there have been no statistical differences in the changes in the marginal bone level between test and control groups. Thus, the risk of apical creeping of the marginal bone level to expose the rough implant surface could be considered low. The exposure of roughened implant surface would enhance biofilm formation and trigger inflammatory response that would jeopardize the marginal bone level stability and the periimplant health [28].

Several factors that would affect marginal bone stability have been examined. All prosthetic rehabilitation has been performed on intermediate abutments with a length higher or equal to 2 mm. A recent meta-analysis has estimated a weighted mean difference in marginal bone loss of −0.30 mm (95% CI = −0.37 to −0.22 mm, *p* < 0.0001), in favor of the implants that were connected to abutments with a height ≥2 mm [29]. More studies are also available and show that the marginal bone stability benefits from the selection of intermediate abutments with a height ≥2 mm [30, 31]. Moreover, the use of intermediate abutments would also save the marginal bone from excessive mechanical stress [32, 33]. Recent experimental study has compared original and non-original abutments and has found that original abutments allowed for better fit and superior mechanical behavior under cyclic loading [34]. The implementation of one abutment-one time protocol and the use of implants with internal connection have also favored marginal bone stability [29].

5.5-mm long implants have shown similar survival rates to longer implants in the context of the current study. There is cumulative and growing evidence to support this finding [3, 5, 35–38]. Several systematic reviewed have contrasted short (<6 mm) with longer implants [3, 5, 35, 36, 38]. Short implants have demonstrated to be a reliable option with high survival rates, marginal bone stability, and low mechanical complications for the short-implant groups. Splinting short implants would allow for an effective distribution of lateral forces and lower mechanical stress at the individual implants, enhancing their survival [37]. Recent reports have shown high implant survival and marginal bone stability when short implants supported single crowns in the short term [36, 39–44]. Long-term follow-up data have shown higher survival risk for short implants (risk ratio: 0.94; 95%CI: 0.90–0.99) compared to longer implants [40]. Another systematic review have also shown that short implants restored with single crown had higher late failure (4%) that short implants restored with fixed partial denture (2%) [45]. It seems that implant position (mandible/maxilla) have no influence on the survival of short implants [41, 45]. Thus, splinting short implants when possible could be advantageous on the long-term performance.

Immediate loading has not jeopardized the survival of short implants in the current study. Several reports have compared immediate loading of short implants with longer implants [46–49]. Weerapong et al. [46] have demonstrated the absence of statistically significant differences between both implants in survival rate and in marginal bone loss. Another study has compared immediate versus delayed loading of short (<8 mm) implants [49]. It has corroborated the absence of statistically significant differences between the immediate and delayed loading groups in implant survival (92.6% vs 97.5%) or marginal bone loss (−0.2 ± 0.8 mm vs −0.1 ± 0.7 mm), respectively. Similarly, immediate loading of short implants in the posterior area have resulted in high cumulative survival rate and marginal bone stability in partially edentulous patients with severe vertical bone atrophy [50]. A meta-analysis has pooled the data of immediately and early-loaded 322 implants with a follow-up up to 10 years and has estimated a pooled survival rate of 91.63% (95% CI: 88–94%), and a mean marginal bone loss effect of 0.52 ± 0.1 mm (*z* = 3.07, *P* < 0.002) [51]. Another meta-analysis has reported that immediate loading of short (<10 mm) implants have not increased the implant failure risk (odds ratio: 1.38, 95% CI: 0.67−2.84, *p* = 0.997, fixed model) compared to longer implants [52].

The design of the current study has minimized the biological variability by the placement of the test and control implants in the same patient and under the same prosthesis, homogenizing the surgical and prosthetic procedures. The study also reflects the daily clinical practice as there has been no influence on the clinical decision of allocating the implant to one site or another. It benefits from the advantages of the prospective design in the systematic collection of data over time. Marginal bone level has been measured in a periapical radiograph and by the same experienced investigator to reduce the inter-observer variability. However, this study suffers from several limitations that should be considered in the extrapolation of its results. One of these limitations is the selection bias as the implant position was not randomized. Another limitation is the short follow-up time that allowed the assessment of implant survival, marginal bone stability and prosthetic complications in the context of 1 year. However, there is a need for clinical data of implants with a length <6 mm to enhance the conclusions of systematic reviews on their clinical performance [6]. The researchers were not blinded to the implant allocation to study groups as they can be differentiated by the implant length, introducing observer bias. The performance of radiographic measurements by a single investigator is a source of systematic bias. Furthermore, the results could be extrapolated to posterior regions and to fixed partial prosthesis, limiting their extrapolation to other clinical scenarios and settings.

## Conclusions

This prospective controlled clinical study supports the use of 5.5-mm long implants in the context of fixed partial prosthesis and posterior regions. No statistically significant differences have been observed in marginal bone stability, implant survival and prosthetic complications compared to longer implants.

## Data Availability

The datasets used and/or analyzed during the current study are available from the corresponding author on reasonable request.
